# High-throughput synthetic rescue for exhaustive characterization of suppressor mutations in human genes

**DOI:** 10.1007/s00018-020-03519-6

**Published:** 2020-04-08

**Authors:** Farah Kobaisi, Nour Fayyad, Eric Sulpice, Bassam Badran, Hussein Fayyad-Kazan, Walid Rachidi, Xavier Gidrol

**Affiliations:** 1 University of Grenoble Alpes, CEA, INSERM, IRIG-BGE U1038, 38000 Grenoble, France; 2 Laboratory of Cancer Biology and Molecular Immunology, Faculty of Sciences I, Lebanese University, Hadath, Lebanon; 3 University of Grenoble Alpes, SYMMES/CIBEST UMR 5819 UGA-CNRS-CEA, IRIG/CEA-Grenoble, Grenoble, France

**Keywords:** Suppressor mutation, Genetic screening, Cell phenotype, Synthetic rescue

## Abstract

Inherited or acquired mutations can lead to pathological outcomes. However, in a process defined as synthetic rescue, phenotypic outcome created by primary mutation is alleviated by suppressor mutations. An exhaustive characterization of these mutations in humans is extremely valuable to better comprehend why patients carrying the same detrimental mutation exhibit different pathological outcomes or different responses to treatment. Here, we first review all known suppressor mutations’ mechanisms characterized by genetic screens on model species like yeast or flies. However, human suppressor mutations are scarce, despite some being discovered based on orthologue genes. Because of recent advances in high-throughput screening, developing an inventory of human suppressor mutations for pathological processes seems achievable. In addition, we review several screening methods for suppressor mutations in cultured human cells through knock-out, knock-down or random mutagenesis screens on large scale. We provide examples of studies published over the past years that opened new therapeutic avenues, particularly in oncology.

## Introduction

Genetic information encoded by the DNA should be preserved and faithfully transmitted across generations. This process is essential for determining the genetic composition of a species. Therefore, any mutation can alter the life of the affected species. Mutations range from nonsense, missense or frameshifts. These alterations’ outcomes can change the functionality of encoded proteins or block their translation generating a diseased phenotype [1].

However, mutation is a double-edged sword. First, random mutations are the basis of evolution and organism adaptation to the environment, an aspect not discussed here [2]. Second, deleterious mutations have a counterpart: suppressor mutations. Assuming a primary mutation creates a diseased phenotype, a new mutation(s) can reverse its effect to generate a wild-type or less severe phenotype and, thus, is defined as synthetic rescue [3]. Synthetic lethality, on the contrary, involves cell death arising from the combination of loss of function mutations in at least two genes where the loss of function in any gene individually does not contribute on its own to cell death. This can be beneficial in identifying tumor vulnerabilities. Suppressor mutations have been found by genetic screening in yeast, flies and worms, enabling the understanding of genetic interactions occurring during development [4, 5]. The existence of secondary mutations may explain how some individuals appear healthy despite harboring disease-causing mutations [6]; while, on the other hand, some patients show resistance to treatments [7]. For example, a secondary mutation in fetal globin genes can ameliorate the effects of β globin gene mutation, modifying the severity of sickle cell anemia [8]. In addition, a mutation in solute carrier family 30 member 8 (*SLC30A8*), which encodes an islet zinc transporter, decreases the risk of diabetes by 65% even in the presence of risk factors such as obesity [9]. The discovery of human suppressor mutations is still an emerging field with little documentation. Suppressor mutations are classified according to whether they are located in the same gene as the primary mutation to be intragenic or not hence extragenic.

With the growth of genome-wide association studies (GWAS), characterization of synthetic rescue mutations in humans would be valuable to comprehend phenotypic outcomes in patients and create new therapeutic strategies based on synthetic rescue. Moreover, due to recent advances in cell phenotype-based screening, it is only logical that such approaches are used for the identification of suppressor mutations. The screening methods rely on CRISPR–Cas9, RNAi, insertional mutagenesis and chemical screens combined with cell phenotype quantification methods [10–13].

## Classification of suppressor mutations

### Intragenic suppression

Intragenic suppression refers to the counteraction of an altered phenotype by a suppressor mutation located in the same gene as the primary mutation [14]. The classification is divided into true and pseudo-revertant mutations.

#### True revertant

**Fig. 1:**
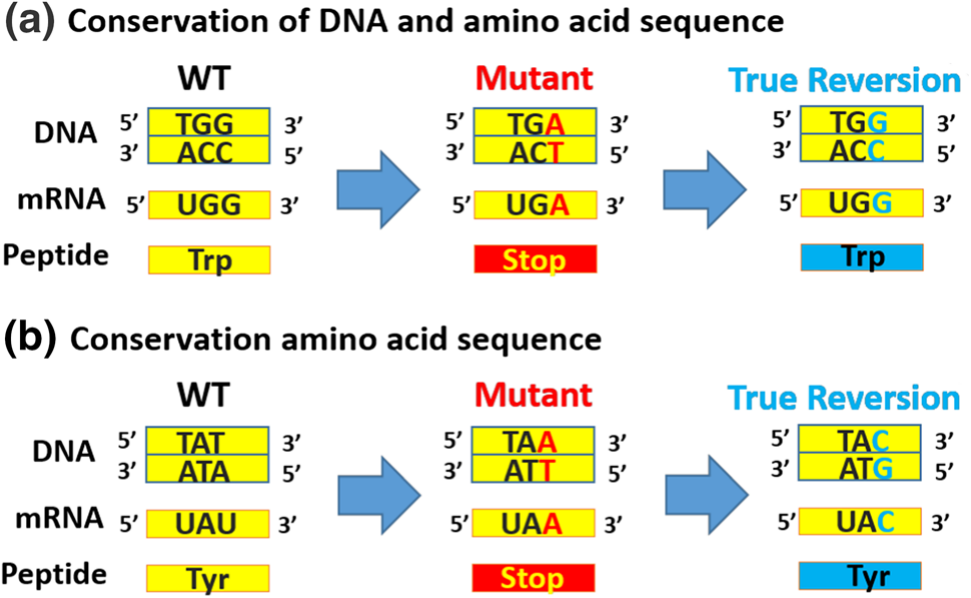
True Revertant Intragenic Suppression. The primary and secondary mutations occur in the same codon and at the same level, the nucleotide level. **a** A mutation that changes a guanine nucleotide into an adenine nucleotide can lead to the conversion of an encoded amino acid from tryptophan into a stop codon. A true reversion mutation can change the sequence back to tryptophan with an adenine mutated to guanine such that tryptophan replaces the stop codon. Thus, the DNA and amino acid sequences are conserved. **b** In another scenario, a mutation of thymine to adenine changes the tyrosine amino acid to yield a stop codon. A true reversion mutation that changes the adenine to cytosine will enable the conversion of the stop codon back to tyrosine, thereby conserving the amino acid sequence alone. *WT* wild type, *Trp* tryptophan, *Tyr* tyrosine

A single base modification caused by a primary mutation is completely reversed to the wild-type sequence due to a suppressor mutation at the same position [15] (Fig. 1a) that restores the wild-type amino acid sequence. However, suppressor mutation does not necessarily restore the same DNA due to the redundancy of the genetic codon (Fig. 1b). This was reported in *Caenorhabditis elegans* by Novelli et al., who found that a glutamine to proline mutation in the collagen processing protease (*dpy-31)* was reverted back to the wild-type phenotype [16].

#### Pseudo-revertant

The function of the gene product will be partially or fully restored even if the wild-type sequence is not restored. The suppressor mutation produces a modified DNA or amino acid sequence that can still carry on the functional or structural characteristics of the wild-type gene [14]. Pseudo-reversion is achieved through various mechanisms.

##### Base modification

**Fig. 2:**
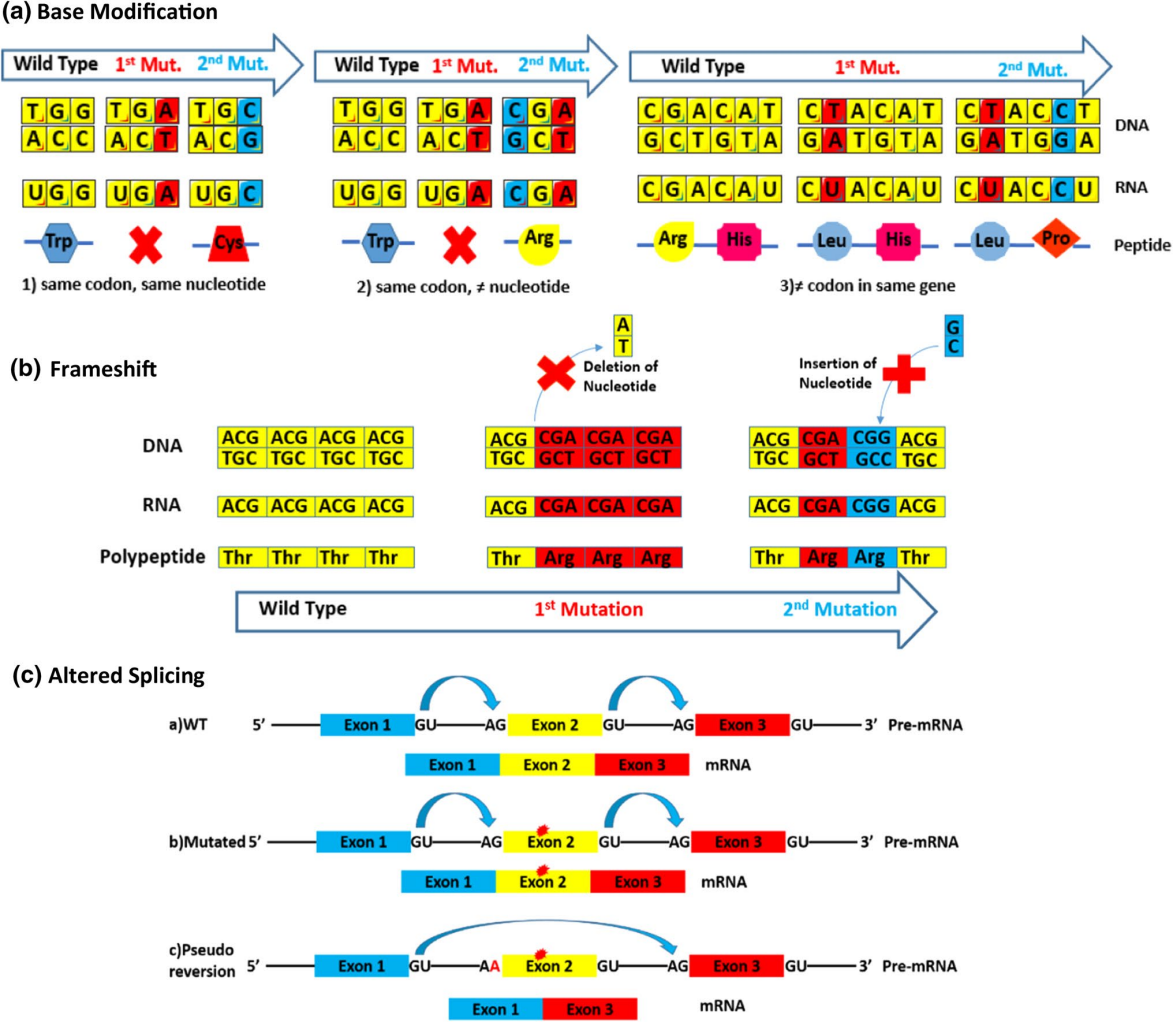
Different forms of intragenic pseudo-reversion. Pseudo-reversion of primary mutation can occur by various types of secondary suppressor mutation. The first is **a** base modification further classified depending on the position of both mutations that can be in the (1) same codon and same nucleotide as the primary mutation resulting in a different translation output, from stop codon to cysteine for example, or (2) same codon but in a different nucleotide to produce arginine instead of a stop codon. In contrast, genes can be mutated at (3) different codons in the same gene to yield the amino acid proline near the mutated leucine. **b** A frameshift secondary mutation can partially restore the reading frame if the primary deletion mutation is counteracted by a suppressor insertion mutation. **c** Finally, altered splicing may mean that exons carrying mutations are skipped. This skipping can occur due to suppressor mutations altering the splice acceptor sites near the mutated exon that enable skipping and hence eliminate the effect of the primary mutation. *WT* wild type, *Trp* tryptophan, *Cys* cysteine, *Arg* arginine, *Leu* leucine, *His* histidine, *Pro* proline

A single base change created can be corrected by another base modification either in the same codon or nearby. This modified gene codes for a different amino acid, but the resulting product can still restore the functionality of the original protein. The different base modifications can occur either in the same codon and the same or different nucleotide or at different codons in the same gene [5] (Fig. 2a). Examples from *C. elegans* include the conversion into proline amino acids created by a primary mutation that changed a glutamine to serine in the *dpy-31* gene. This reversion prevents the lethality induced by loss of function mutation of the latter gene [16]. Furthermore, several second-site missense mutations in the cell interaction *glp-1* gene promote the reversion of the primary temperature-sensitive missense mutation [17].

##### Frameshift mutation

They involve changes in the reading frame due to the insertion or deletion of nucleotides. Addition of a nucleotide that results in a detrimental change to the reading frame can be restored by a deletion mutation nearby that restores the reading frame [18] (Fig. 2b).

##### Altered splicing

Multiple splice variants genes demonstrate altered splicing where a mutation in one of the alternatively spliced exons can be suppressed by a mutation to the acceptor site near said exon omitting it (Fig. 2c). The perlecan-encoding gene *unc-52* exhibits altered splicing that reverts the effect of the primary mutation causing defects in myofilament assembly and in the attachment of the myofilament lattice to the muscle cell membrane [19]. This mechanism is utilized for the treatment of Duchenne Muscular Dystrophy in which antisense oligonucleotides mimic altered splicing suppressor mutation by biding to the site of mutation and induce the skipping of the mutated exon [20].

### Extragenic suppression

The rescue of the mutant phenotype by a suppressor mutation located in a different gene is termed extragenic suppression. This involves either informational or functional suppression.

#### Informational suppression

Informational suppression is allele-specific and gene-nonspecific [21] mutations involved in the transmission of genetic information from DNA to protein. They involve mutations that alter the machinery of DNA transcription, RNA processing, or protein translation. The genes responsible for stabilizing mRNAs or proteins can also influence the mutations that fall into this category where this stabilization may compensate for the poor functionality of a mutated product. The two genes involved in information suppression do not share a close functional relationship in a given developmental or pathological process.

##### Nonsense suppressor tRNA: translational suppression

**Fig. 3:**
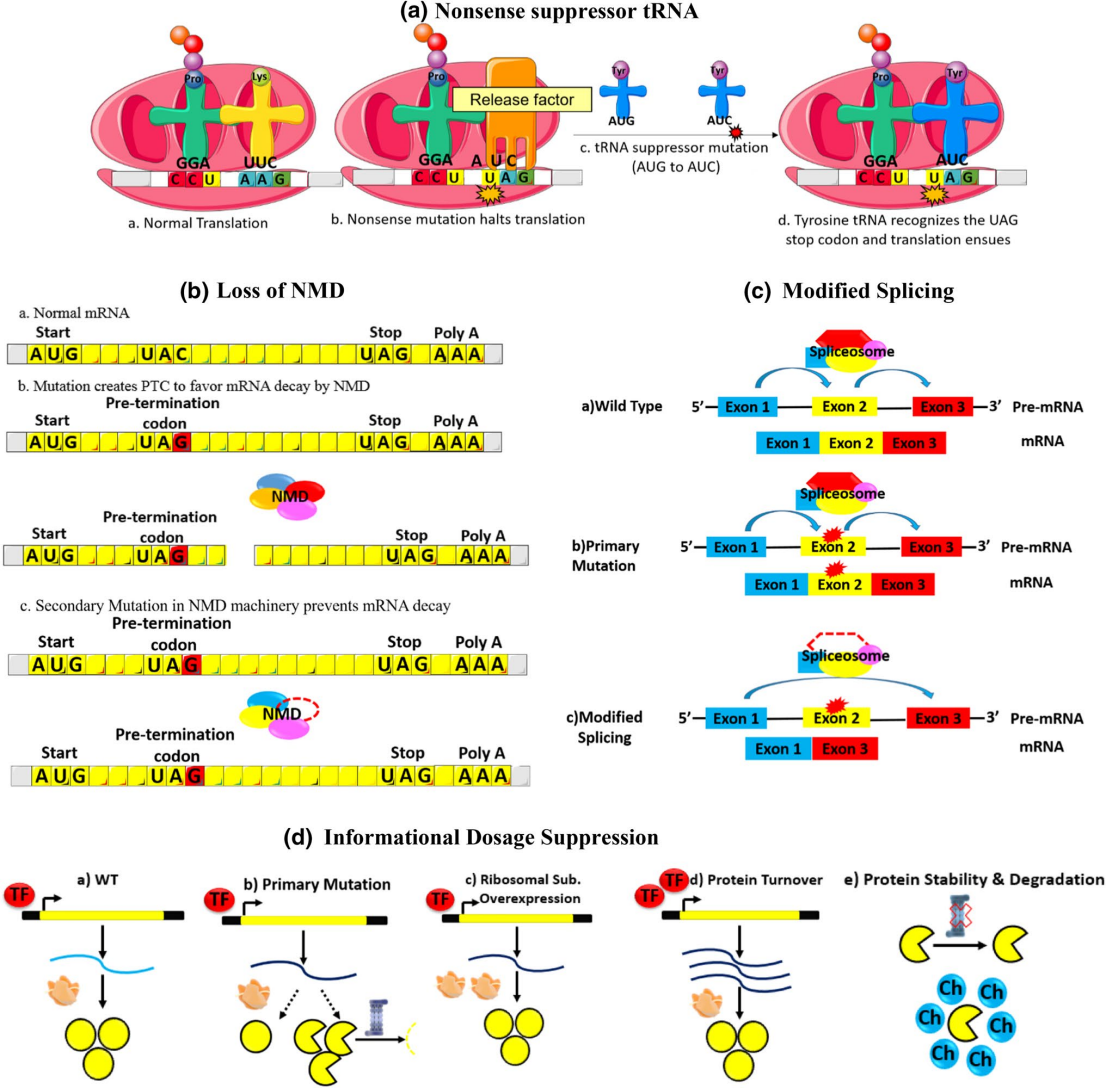
Extragenic Informational Suppression. Informational suppression deals with any suppressor mutations that alter the transmission of genetic information form DNA to protein. (a) Nonsense suppressor tRNA. tRNA with the anticodon 3′UUC 5′ carries a lysine amino acid to add to the growing polypeptide when it encounters the codon 5′AAG3′. Mutation of this codon from 5′AAG3′ to 5′UAG3′ results in a stop codon. However, a tRNA suppressor mutation that mutates a 3′AUG5′ anticodon to 3′AUC5′ results in a tRNA that still carries the same amino acid tyrosine but can recognize the 5′UAG3′ stop codon and prevent the termination of translation. (b) Loss of NMD. A nonsense mutation can cause the mRNA to carry a premature termination codon (PTC) that targets it for degradation by NMD. However, a suppressor mutation in the NMD machinery can block degradation and prevent protein loss. (c) Modified splicing involves skipping the exons that carry mutations. This phenomenon occurs due to suppressor mutations that alter the spliceosome, which enables the mutated exon to be skipped, thus eliminating the effect of the primary mutation. (d) Informational dosage suppression. Primary mutations can either decrease the expression or code for modified proteins that are subjected to proteasomal degradation. Suppressor mutations can increase the expression of the mutated protein by overexpressing either ribosomal subunits or transcription factors. Another mechanism deals with protein stability and degradation whereby a mutation in proteasomes can protect the mutated protein from degradation, or the protein can be rescued by overexpressed chaperone proteins that stabilize the mutated protein. tRNA: transfer RNA, Pro: proline, Lys: lysine, Tyr: tyrosine, NMD: nonsense mediated mRNA decay, PTC: premature termination codon, TF: transcription factor, CH: chaperon

Nonsense mutations result from base modifications creating any of the three nonsense codons: UAA (ochre), UAG (amber), or UGA (opal) leading to termination of translation, hence partial or complete loss of gene function. However, suppression of this mechanism is enabled by nonsense suppressor tRNAs with anticodons that recognize stop codons and add amino acids at such sites to partially or fully restore protein function (Fig. 3a). A nonsense suppressor tRNA class with a mutation in the anticodon loop exists for the recognition of each type of the three stop codons [22]. For example, tRNA with a 3′AUG5′ anticodon carries a tyrosine amino acid that is added to the growing polypeptide when it encounters the 5′UAC3′ codon. Mutation in this anticodon from 3′AUG5′ to 3′AUC5′ results in a tRNA that still carries tyrosine but can now recognize the amber stop codon 5′UAG3′ and add this tyrosine to the growing polypeptide chain instead of terminating translation (Fig. 3a). One downside of these mutated tRNAs is that they can no longer recognize their wild-type codons. However, tRNA genes are available in multiple copies across the genome; therefore, other copies can still code for the unmutated tRNA [23]. O’Neill et al. showed the presence of mutated tRNA genes by screening a human DNA library cloned in a bacteriophage with an opal suppressor tRNA probe [24].

##### Loss of NMD

NMD refers to “nonsense mRNA decay” initiated by the presence of a premature termination codon (PTC) 50–55 nucleotides upstream of the final exon–exon junction and leads to the downregulation of the transcript via its degradation [25]. However, the absence of NMD ensures a longer half-life for the transcripts carrying this PTC, increasing the chance that they will be translated. This loss can be mediated by mutations at the NMD level, including mutations in mRNA decay-associated *SMG* kinase genes, enabling the translation of the mutated mRNA and, thus, suppressing the effects of the primary nonsense mutation [5] (Fig. 3b). It should be noted that increasing the translation of disease-associated mRNA is utilized for the treatment of certain genetic disorders, including Duchenne muscular dystrophy, rendering the loss of NMD an appealing suppressor mechanism [26]. Loss of NDM was first identified in *C. elegans* following the creation of nonsense mutations by forward mutagenesis screens that yielded mutations in genes associated with NMD [27].

##### Modified splicing

Unlike the altered splicing in pseudo-revertant mutations, the effect of the primary mutation is suppressed by mutations at the level of the splicing machinery (Fig. 3c). In *C. elegans*, loss-of-function mutations in *smu* genes encoding proteins homologous to mammalian spliceosome-associated factors lead to enhanced exons skipping, including the skipping of exons with primary mutations [28]. The mutated perlecan gene *unc-52* is reportedly suppressed by loss-of-function mutations in the *smu-1 and smu-2* genes in addition to its suppression by altered splicing.

##### Informational dosage suppression

Dosage suppression involves elevated gene expression rather than a gene mutation to suppress the altered phenotype (Fig. 3d).

- *Overexpression of ribosomal subunits* The overexpression of ribosomal subunits has counterbalancing outcomes. Certain studies found a correlation between genetic fitness and ribosomal composition, suggesting a role for ribosomal subunit stoichiometry in regulating translation or affecting the progression of the cell cycle [29]. However, another study attributed a different role for these proteins, suggesting that they are possible chaperones that increase the stability of the proteins harboring mutations and shielding them from degradation [30].
- *Protein turnover* The overexpression of transcription factors leads to increased transcription of mutated genes. This increased gene expression increases the likelihood that the upregulated RNA will be translated [31].
- *Modulating protein stability or degradation* Mutated mRNA can be translated to yield unstable proteins prone to degradation. Interfering with this degradation or stabilization of proteins can increase their half-lives, which may enable them to partially function, depending on the severity of the original mutation. J. Van Leeuwen et al. demonstrated that a genetic mutation in ubiquitin protein kinase *san1* annuls its role in targeting proteins’ hydrophobic residues for proteasomal degradation [6]. In addition, overexpression of chaperones that stabilize the mutant protein increases the pool of active mutated proteins [31].

#### Functional suppression

Mutations can modulate the function of gene products by affecting their posttranslational modifications, localization or their interaction with either activators or inhibitors. Functional suppressors can help substitute or restore this modulated function via the alteration of proteins functionally related to the primary proteins [32]. Different classes of functional suppressors exist, and they are all gene specific.

##### Epistasis

**Fig. 4:**
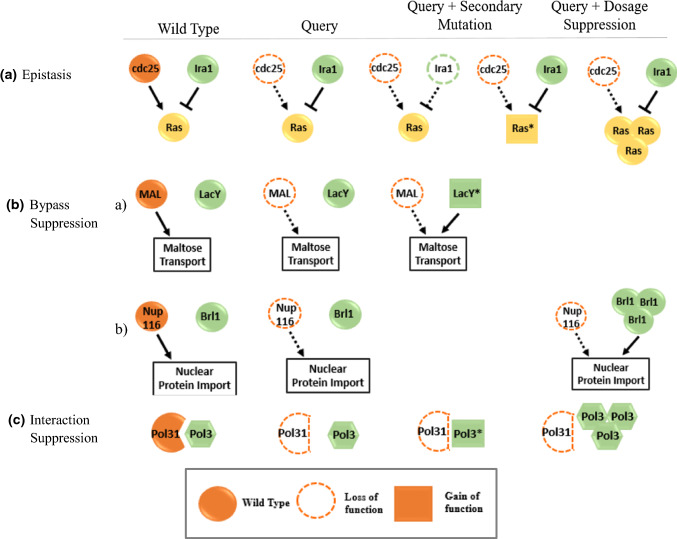
Functional suppression: epistasis, bypass and interaction suppression. **a** Epistasis describes a suppressor mutation in a protein belonging to the same pathway as the protein that was primarily mutated. For example, a mutation in cdc25, a guanine nucleotide exchange factor, prevents downstream Ras activation but can be suppressed by either a secondary mutation in GTPase activating protein Ira1, a known inhibitor of Ras activation, or by a gain-of-function mutation in Ras itself. Dosage suppression was also recorded in which Ras overexpression can suppress the mutation of cdc25. **b** Bypass suppression involves two mutated genes that belong to different pathways that are functionally related. Mutations altering the specificity of lactose permease (LacY) permit the transport of maltose, despite the mutation to maltose permease (MAL). Another example is that of the overexpressed BRL1 involvement in changing the nuclear membrane composition to suppress the inhibited nuclear protein import that was induced by a mutation in Nup116. The latter mechanism can be categorized as dosage suppression. **c** Interaction suppression involves mutations in proteins that belonging to the same complex. For instance, the mutation in the DNA polymerase delta subunit Pol31 is suppressed by either the mutation in the catalytic subunit Pol3 of DNA polymerase or by its overexpression. *Cdc25* guanine nucleotide exchange factor, *Ira1* GTPase activating protein, *LacY* lactose permease, *MAL* maltose permease, *BRL1* Nucleus export protein, *NUP116* nucleoprotein 116

Signal transduction requires sequential steps initiated by receptor activation, second messengers, effectors and transcription factors. The modulation of one step can hinder message transmission. Epistasis involves additional mutations in a different step upstream or downstream, but in the same pathway, that can restore protein functionality [33]. This occurs in regulatory switch pathways alternating between “on” and “off” state where the primary and suppressor mutation often have antagonistic effects. For example, in the sequential pathway of signal → protein 1—I protein 2—I protein 3, the signal activates protein 1, which in turn inhibits protein 2 to abolish its effect in the inhibition of protein 3, thus rendering protein 3 active. A primary mutation that disrupts the function of protein 1 will block this pathway due to the constitutive activation of protein 2. However, an additional suppressor mutation to protein 2 enables the activation of protein 3, although in a constitutive manner. The two mutations in this example scenario are loss-of-function mutations. Another possible mechanism for epistatic suppression in the same example pathway is the combination of a primary loss-of-function mutation in protein 1 and a simultaneous gain-of-function suppressor mutation in protein 3, reestablishing the pathway. Mutation in yeast cdc25, which encodes the guanine nucleotide exchange factor, prevents downstream Ras activation but can be suppressed either by a secondary loss of function mutation in GTPase activating protein Ira1, a known inhibitor of Ras activation, or by a gain-of-function mutation in Ras itself (Fig. 4a). Dosage suppression was also recorded where Ras overexpression suppresses cdc25 mutation [34]. In mice, the functional loss of Mdm2, a Trp53 negative regulator, can be suppressed by the functional loss of P53 [35].

##### Bypass suppression

Bypass suppression genes belong to different pathways biochemically or genetically parallel. Here, the alteration of protein activity in a pathway is compensated by one of the two modifications of an alternative protein in a related but different pathway [36]. The first involves bypassing of protein 1 loss of functionality by the alteration of the specificity of protein 2 such that the alternate pathway is rewired to perform the function of protein 1. In *E. coli*, mutations the alter the specificity of lactose permease permit the transport of maltose, despite the presence of a mutated maltose permease [37] (Fig. 4b).

Moreover, in yeast, the reduction of the mitochondrial membrane potential caused by the absence of mitochondrial ribosomal protein Mrpl3 is restored by the gain-of-function mutation in Atp1, an ATP synthase subunit [6]. The other model can be classified as both bypass and dosage suppressor because the amount of the alternate protein is the factor responsible for the compensation of the modified activity. The overexpression of protein 3 can overcome protein 1 mutation despite being of low constitutive activity at normal expression rate [38]. For instance, the overexpression of BRL1 involved in changing the nuclear membrane composition can suppress the inhibited nuclear protein import caused by a mutation in NUP116 [39] (Fig. 4b).

##### Interaction suppression

Interaction suppression affects proteins belonging to the same complex and it is allele- and gene specific [14]. Mutations that change the shape or binding site of one protein prevent its complexation abrogating the complex’s function. However, a suppressor mutation that creates a compensatory shape change in the second protein can help restore the lock-and-key interaction [40]. Another mechanism relies on the overexpression of another complex subunit that increases the recruitment ability of the mutated component without a compensatory change in the shape of the secondary protein [41]. The overexpression mass action will kinetically drive the formation of the complex, regardless of the decreased binding constant. Moreover, overexpression of a paralog can also compensate for this loss [42]. Finally, a gain-of-function mutation in one of the remaining complex subunits can reinstate the complex function by either stabilizing it or completely substituting the function of the originally mutated component [43]. In yeast, mutation in the DNA polymerase delta subunit Pol31 is suppressed by either the mutation in the catalytic subunit Pol3 of the DNA polymerase or by its overexpression [44] (Fig. 4c). Another example from yeast involves both Cdc2 kinase and Cdc13 cyclin, for which a suppressor mutation that introduces an additional contact surface between the two proteins help reestablish complex formation [45, 46].

## High content screening for suppressor mutations

The relationship between genotype and phenotype has long been studied by genetic screens introducing genetic perturbation into cell or organism to investigate their effects on phenotype. Suppressor screens go one step further to identify modulators of an altered phenotype with an inborn genetic abnormality in an attempt to overcome the outcome of primary mutation.

In silico suppressor screen was performed by Das Sahu et al. It aimed to identify synthetic rescue (SR) mediators of resistance to immunotherapy by analyzing tumor transcriptomics and survival data of cancer patients. Several SR interactions in cancer cells resistant to therapy have been reported, some of which are rescuers of DNA methyl transferase (DNMT), which renders the cells resistant to killing by the DNMT1 inhibitor decitabine [7]. Another algorithmic-based screening was carried out by Motter et al. who predicted secondary mutations in metabolic pathways reversing the growth defect of a primary metabolic one [47]. The utilization of this same approach to compare transcriptomes from patients with similar primary mutations but differing symptoms can help identify secondary suppressor mutations and synthetic rescuers. Nonetheless, other conventional screening approaches include CRISPR–Cas9, RNAi, insertional mutagenesis and chemical-based screening (Table 1).

**Table 1:** Pros and Cons of the different mutagenesis/gene expression alteration methods

Screening technique	Pros	Cons
CRISPR–Cas9	Induces irreversible alteration into the DNA that is transmitted to progeny	PAM sequence restrictions
	Enables alterations in transcribed and untranscribed regions	Low efficiency of HDR relative to NHEJ
	Engineers versatility in Cas protein	
RNAi	Target genes can be identified immediately	Can partially suppress genes such that they are limited to knocking them down
	RNAi can be chemically modified	Exhibits off-target effects
	RNAi can be used as a therapeutic agent	Limited to transcribed regions
Insertional mutagenesis	No need for complex library design	Random insertion
		Sequencing is necessary
Chemical	No need for complex delivery techniques	Requires time-consuming target identification

### CRISPR-based screening

**Fig. 5:**
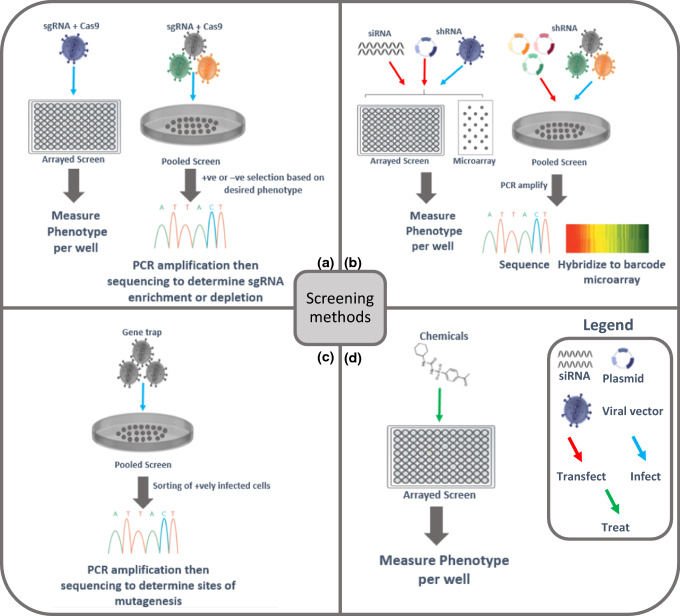
Screening formats and methods. **a** CRISPR–Cas9 screening requires the delivery of the sgRNA and Cas-9 through viral vectors to infect cells in either an arrayed format, followed by the detection of phenotypic changes in each well or in a pooled format in which cells are positively or negatively selected based on the phenotype of interest, followed by PCR amplification and sequencing for the identification of the sgRNA that is enriched or depleted. **b** RNAi screening can be done by transfecting siRNA or shRNA plasmids or by transducing cells with shRNA viral vectors. These three forms can be used in the arrayed or micro arrayed format to target one gene per well/spot and measure readout. The pooled format utilizes shRNA in plasmids or viral vectors. The readout can be assessed by PCR amplification and sequencing of shRNA, or the barcode containing shRNA can be hybridized to the barcode microarray. **c** Insertional mutagenesis requires the transduction of cells in a pooled format with viral vectors carrying a gene trap cassette. Once the latter is inserted, the cells will be sorted if they are positively transduced and carry the phenotype of the insert. PCR amplification and sequencing will then enable the identification of sites of mutagenesis. **d** Chemical screens are initiated by treating cells in an arrayed format with one drug per well. The difficulty to track individually every drugs’ effect and the possibility of interaction between drugs render it impossible for this assay to be carried out in a pooled format. Different phenotypic outputs can be assessed after the arrayed format treatment. *CRISPR* clustered regularly interspaced short palindromic repeats, *sgRNA* single guide RNA, *siRNA* short interfering RNA, *shRNA* short hairpin RNA

CRISPR–Cas9 screens can be conducted in either arrayed or pooled formats. In both cases, cells harboring a particular mutation are subjected to knock-out of different genes to assess the desired phenotype. In the arrayed format, a single gene is deregulated in each well and different phenotypes can then be investigated, such as death versus survival, or phenotypical changes at the single cell level [48]. In contrast, a pooled screen offers more high-throughput characteristics and delivers a massive library to cells that are then sorted based on a particular phenotype of interest. This sorting can be either a positive selection in which the perturbations that enable survival are enriched, or negative sorting where targets deplete cells from the population. The effects of genetic perturbations are determined by comparing the sgRNA profile. The latter selection is more laborious as it requires a highly sensitive readout. Nonetheless, following both selection techniques, DNA is extracted and PCR amplification of the sgRNA encoding regions is performed to be further on sequenced and mapped. This process identifies either the enriched or depleted genes [49] (Fig. 5a). It should be noted that CRISPR–Cas9 screens have recently evolved such that they can be used not only for knockdown screens but also for loss-of-function or even activation screens due to the different possible modifications to sgRNA or to Cas9, such as those that disable some of its activities and fuse it with various effectors [50]. Moder et al. aimed to find a suppressor mutation enhancing the viability of cells deficient in the Fanconi anemia pathway. They generated their own disease model by introducing a frame-shift mutation in FANCC by CRISP–Cas9, which led to the loss of its protein expression [51]. Loss of FANCC renders the cells hypersensitive to cross-linking agents such as mitomycin C (MMC). The cells were transfected with a pooled genome-scale CRISPR-knockout library and then selected with MMC to identify suppressors that rescue ΔFANCC hypersensitive phenotype. Moder et al. successfully identified several members of the BLM complex that is part of a multienzyme DNA helicase and bridged to the FA complex by FANCM [52].

Similarly, MUTYH gene suppression was determined to alleviate photosensitivity in cells harboring mutations in the nucleotide excision repair gene XPA. This mechanism was discovered through the combined chemical and CRISPR–Cas9 effects on a CRISPR–Cas9-created XPA-deficient cell model [53].

### RNAi-based screening

Different forms of interfering RNAs are microRNA (miRNA), small interfering RNA (siRNA) or short hairpin RNA (shRNA). miRNAs are generally not used in screens due to their partial complementarity that permits them to target several transcripts [54]. siRNAs are synthetically produced and can be introduced to the cells directly or within a plasmid as shRNA.

When performing RNAi-based screens, one should first identify which type of interfering RNA best suits the screen. If short-term silencing is required, siRNA libraries are the right choice as they do not undergo replication and are diluted upon cell division. siRNA libraries are mainly delivered to cells by lipid particles [55]. Otherwise, for long-term and stable silencing, shRNA vectors that can be integrated into the genome and copied to the progeny are used. The utilized vectors could be either nonviral plasmids or retroviral, adenoviral, or lentiviral vectors [56].

Similar to CRISPR–Cas9-based screens, RNAi screens are performed in two formats. Both siRNA and shRNA vectors can be utilized in the arrayed format each targeting one gene per well [57]. Pooled format is carried out with shRNA transfected or transduced in a large cell population then selected according to a particular phenotype. This process is followed by PCR amplification and sequencing of the enriched shRNA. Additional DNA sequences can also be added to the vectors to act as barcodes easing the identification of hits. This technique can also be used in a microarray format in which normal and mutant cell populations are transduced with barcode-containing vectors that are linked to two distinct fluorochromes, each unique to a population. The hybridization of DNA to the barcode probes on a microarray will favor the comparison between the shRNA profile of the normal and mutant cells [58] (Fig. 5b). Microarray is not only used for assessing the output but can also be a screening format on its own. siRNAs, shRNA plasmids or even shRNA viral vectors can be arrayed on slides as individual drops, each targeting a specific gene. Cells can then be plated directly. After identifying the positions where a preferred phenotype is located, it can be simply linked to the target gene [59].

Luo et al. utilized a pooled genome-wide retroviral shRNA screen. The barcode-incorporated shRNA vectors were transfected into normal and colorectal cells with KRAS activating mutation followed by microarray-based comparison of shRNA profiles. They obtained several shRNA with anti-proliferative activity depleted from the population [60].

### Insertional mutagenesis-based screening (Gene trap)

A gene trap cassette contains a promoterless reporter expressed only when inserted in the correct orientation in a transcriptional unit. It also contains one or several splice acceptor sites that render their expression conditional to the insertion of such cassette into introns or exons. Splicing enables the fusion of splice donor sites with the reporter splice acceptor creating transcriptional fusion and, thus, a fusion protein. Some insertions render the protein nonfunctional [61]. The procedure for insertional mutagenesis screening starts with the transduction of gene trap viruses. Expression of a reporter gene in the cassette allows the identification of the transduced cells to be FACS isolated. Phenotypic sorting is then performed to enrich the mutations favoring survival. The mutagenesis position is then determined by sequencing [62] (Fig. 5c).

Moder et al. performed a gene trap screen in parallel with the CRISPR–Cas 9 screen described earlier. They identified the loss of NAD(P)H:quinone oxidoreductase 1 (NQO1) as a possible suppressor mutation that alleviated the hypersensitivity of ΔFANCC HAP1 to MMC. Furthermore, BLM loss identified by CRISPR–Cas9 was also identified in the insertional mutagenesis screen [51]. A similar screen by Velimezi et al. led to the identification of *USP48*, that has a synthetic rescue interaction with FA genes [63]. The loss of *USP48* enhanced the recruitment of homologous recombination proteins RAD51 and BRCA1 and reduced chromosomal instability.

### Chemical-based screening

Chemical screens can be either phenotypic or target based. Phenotypic screening involves the treatment of cells with molecules that enable the identification of hits that mediate the formation of a desired phenotype. This methodology allows for selecting compounds directly active in the cells. In contrast, target-based screens are mostly focused on purified protein(s) and how the treatment with different compounds can affect it (their) functions or interactions. The limitation of the target-based method has to do with the fact that certain compounds may not be active within the cell or may interact with multiple targets depending on the cell context, thus abolishing the paradigm of one compound for one target [64]. Chemical screening consists of cell seeding followed by compound treatment and incubation (Fig. 5d). Screen variables include compound concentration and incubation duration. Screening is conducted in an arrayed format with different possible readouts [65].

The biggest drawback for cell-based chemical screening is target identification, especially for newly created active compounds. Several target deconvolution techniques have been developed for phenotypic screens, including affinity chromatography, activity-based protein profiling, analysis in silico or expression cloning [66].

Alli et al. conducted a chemical screen on a BRCA1-mutated breast cancer cells deficient in base excision repair (BER). To visualize the reversal of the BER, the cells were transduced with an adenovirus coding for ODD and containing GFP. The expression of the reporter is only mediated if the damage is repaired due to treatment, signifying that the compound restored the repair process. The compounds identified were the FDA approved acetohexamide and benserazide [67] previously used in the treatment of diabetes and Parkinson; therefore, rerouting their use to potentially generate a new outcome would be relatively easy. In another screen, CRISPR–Cas9-generated XPA-deficient HAP1 cell lines were treated with a library of 290 FDA-approved drugs, and the hits corresponded to drugs that alleviate the photosensitivity and enable the repair of DNA damage following UV irradiation. Acetohexamide was identified for its NER repair enhancement capacity [53].

## Examining drug resistance by functional genetic screens

The development of drug resistance in the course of treatment is a common issue that hinders patient recovery. For instance, pancreatic ductal adenocarcinoma with oncogenic KRAS mutation exhibits resistance to gemcitabine treatment mediated by the gain of function of DCLK1. The targeting of the later protein with anti-DCLK1 antibody inhibits in vivo tumorigenesis [68]. Thus, the identification of resistance mechanisms and mediators is of great importance. The latter seems possible with advances in screening technologies, especially in the fields of RNAi and CRISPR–Cas9. The most robust of such screens are survival-based positive selection screens. They are mediated by the introduction of either pooled shRNA or gRNA followed by the treatment with the drug in question for which the effect is hindered due to resistance. The identification of enriched sh/gRNA in these resistant cells enables the determination of possible targets for inhibition [69]. It is worth noting that the arrayed format of these screens is also effective for identifying resistance targets. For example, in a synthetic lethal experiment, treatment with AKT kinase inhibitor combined with a kinome RNAi library screening helped to identify kinases involved with AKT for the mediation of survival, including inositol polyphosphate multikinase (IPMK). The latter can be targeted in combination with inhibited AKT to favor synergistic synthetic lethality [70]. Moreover, the involvement of PRC2 complex suppression in the mediation of resistance to BET inhibitors in Acute Myeloid Leukemia was also discovered by carrying out a pooled shRNA library targeting 626 chromatin-associated murine genes. The effect was mediated indirectly via the remodeling of regulatory pathways favoring the transcription of genes like *Myc *[71]. CRISPR–Cas9-based genome-wide screen in melanoma cells co-cultured with cytotoxic T cells identified the inhibition of members of the SWI/SNF chromatin remodeling complex, as sensitizers for T-cell-mediated tumor killing and reversion of immunotherapy resistance [72]. Moreover, gene trap screens have also been utilized in synergy with chemical treatment. Bigenzahn et al. reported, after introducing a gene trap cassette in BCR-ABL^+^ leukemia cells, that the inactivation of LZTR1 enables resistance against several BCR-ABL inhibitors due to increased RAS activity [73]. One final approach for deciphering resistance mediators can be via the utilization of sequencing. RNA sequencing of prostate circulating cancer cells from patients treated with androgen receptor inhibitors compared to untreated counteracts revealed the involvement of non-canonical Wnt signaling in resistance facilitation [74].

## Conclusion

Suppressor mutations have opened a new gateway for therapy. Instead of fixing mutations directly through genetic engineering, it is possible to find new targets whose suppression rescue diseased phenotype. Suppressor mutations can range from second-site mutations to those that modify alternate pathways to compensate for loss of protein functionality. Discovery of suppressors started in yeast, flies, *C. elegans* and continues to be investigated in humans. For instance, a recent genetic study, “The Resilience Project,” focuses on screening a seemingly healthy population of people for disease-associated variations. Once identified, these resilient individuals will undergo further genetic and clinical characterization for the identification of disease suppressors [75]. Another screening-based project is Project Achilles that utilizes RNAi and CRISPR to silence individual genes in genomically characterized cancer cells to identify genes affecting survival by algorithms [76]. Nonetheless, the path for suppressor discovery is paved with advances in screening methods and readout analyses. As demonstrated by the limited number of examples described above, large-scale screening procedures offer a way to generate an exhaustive repertoire of synthetic interactions and even synthetic lethal interactions in humans. Such an exhaustive characterization would be valuable for understanding variance in patients carrying same deleterious mutations and resistance to cancer drug treatment [7]. Finally, a better prediction of synthetic rescue mediators in the human genome will open new therapeutic avenues whereby inhibition of a given protein may restore a wild-type phenotype to normalize a pathologic state.
